# Supplementary material for: Minispheroids as a Tool for Ligament Tissue Engineering: Do the Self-Assembly Techniques and Spheroid Dimensions Influence the Cruciate Ligamentocyte Phenotype?
Source: Int J Mol Sci. 2021 Oct 12;22(20):11011. doi: 10.3390/ijms222011011 (PMC8537246; doi:10.3390/ijms222011011)
Supplement: Supplementary file 1 [file ijms-22-11011-s001.zip › ijms-1373072-SI.pdf]

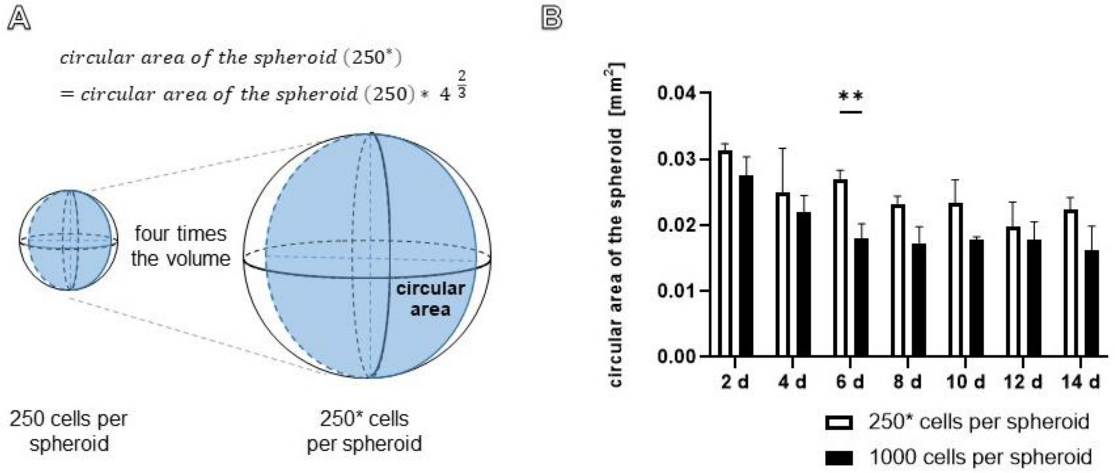

Figure S1. Comparison of the measured circular area of the spheroids with 1000 cells with a hypothetical 4 times volume expansion (250*) calculated for the spheroids with 250 cells documented over 14 days using the spheroid plate. **A**: Schematic representation of the hypothetical 4 times volume expansion. **B**: comparison of 250* and 1000 cells per spheroid. Statistics: n = 3 independent experiments were performed with cells of three different donors. Two-way ANOVA (Bonferroni's multiple comparisons test) for comparison between 250* and 1000 cells per spheroid. p values: **< 0.01 (**B**).
